# Psychosocial Working Conditions, Workplace Violence, and Mental Health Outcomes Among Seafarers

**DOI:** 10.1177/00469580261483999

**Published:** 2026-09-07

**Authors:** Subash Thapa, Lisa Loloma Froholdt, Santosh Giri, Jesper Bo Nielsen

**Affiliations:** 1 Rural Health Research Institute, 1109 Charles Sturt University, Orange, Australia; 2 Research Unit for General Practice, 6174 University of Southern Denmark, Odense, Denmark; 3 Research Unit for Maritime Health and Technology, 6174 University of Southern Denmark, Odense, Denmark

**Keywords:** mental health, seafarers, sexual assault, vulnerable populations, workplace violence

## Abstract

**Introduction:**

The maritime sector is moving toward more diverse crew compositions. While diversity-oriented crewing models may improve safety, inclusion, and team functioning, they may also introduce new vulnerabilities if organizational systems are insufficiently prepared. This study investigates the prevalence and occupational determinants of depression, anxiety, and suicidal ideation among seafarers, with a focus on psychosocial working conditions.

**Methods:**

We conducted a cross-sectional study of 1,046 seafarers from an international fleet using an online questionnaire. Anxiety, depression, and suicidal ideation were assessed using validated self-reported measures. Multivariable logistic regression analyses examined associations between mental health outcomes and occupational, interpersonal, and psychosocial factors.

**Results:**

The results showed that the prevalence of anxiety, depression, and suicidal ideation was 9.2%, 7.5%, and 4.7% respectively. Exposure to psychological violence only was associated with significantly higher odds of anxiety (OR = 2.07; 95% CI: 1.08–3.96) and depression (OR = 2.30; 95% CI: 1.18–4.49), while exposure to combined psychological and physical/sexual violence further elevated the risk of anxiety (OR = 6.92; 95% CI: 2.11–22.65), depression (OR = 5.53; 95% CI: 1.64–18.67), and suicidal ideation (OR = 4.32; 95% CI: 1.03–18.17). Having a fair distribution of workload was associated with lower odds of depression (OR = 0.42; 95% CI: 0.23–0.79) and suicidal ideation (OR = 0.39; 95% CI: 0.19–0.82), while reporting good working relationships with crew members reduced the odds of depression (OR = 0.35; 95% CI: 0.13–0.95) and anxiety (OR = 0.29; 95% CI: 0.10–0.78). High perceived social support was protective against depression (OR = 0.34; 95% CI: 0.13–0.85).

**Conclusion:**

Mental health outcomes are associated with workplace violence, workload distribution, working relationships, and social support. Interventions targeting leadership, bullying, workload distribution, psychological safety and reporting systems are needed.

## Introduction

Mental health conditions, including anxiety, depression, and suicidal ideation, are increasingly recognized as a major occupational health challenge in the maritime industry. Seafarers often experience higher rates of mental disorders compared with shore-based workers, reflecting the cumulative effects of isolation, long voyages, extended work hours, and separation from family and social support networks.^1,2^ The unique working conditions at sea, where crew members live and work in the same confined environment for months, create a continuous exposure to occupational stressors with limited opportunities for psychosocial recovery.^3^ These conditions contribute to substantial psychological strain and elevate the risk of mental health conditions, which may compromise both individual well-being and vessel safety.^4,5^

The working environment in the shipping industry is fundamentally distinct from onshore workplaces. Seafarers cannot leave work at the end of the day, enjoy regular weekends off, or maintain close daily contact with their families. These structural conditions are inherent to seafaring and are not readily modifiable. As a result, understanding how psychosocial working conditions influence seafarers’ mental health is critical for improving well-being and ensuring safe and sustainable maritime operations.

### Literature Review

Existing research indicates that seafarers have higher rates of mental health problems compared with shore-based workers, largely due to cumulative occupational stressors and constrained living conditions.^4,5^ Nor do seafarers have access to health services comparable to those available to their counterparts ashore.^6^ While significant progress has been made in improving physical safety and reducing workplace injuries in the maritime sector, advancements in addressing psychosocial risks and mental health have been more limited.^4,7-9^

A growing body of evidence highlights the importance of psychosocial working conditions in shaping mental health outcomes among seafarers. Workplace bullying, harassment, long working hours, high job demands, and poor leadership have been consistently linked to adverse mental health conditions among seafarers.^1,7,10-12^ This indicates that improvements in physical safety have not been matched by equivalent progress in psychosocial working conditions.^3,13^ Conversely, supportive leadership and strong crew relationships can act as protective factors, buffering the psychological impact of adverse work conditions.^7,11^

The maritime work environment may further intensify these psychosocial risks. Long voyages, hierarchical structures, and culturally diverse crews can contribute to interpersonal conflicts, social isolation, and unequal power dynamics.^4,5,14^ These challenges may be particularly salient in diversity-crewing contexts, where gender, culture, and experience influence workplace interactions and exposure to psychosocial hazards.^11,14^ In parallel, increasing attention has been paid to diversity, equity, and inclusion within the maritime sector. The International Maritime Organization (IMO) has recently promoted initiatives to increase women’s participation, including *the Handbook on Gender Mainstreaming in the Maritime Sector*, which provides practical guidance for integrating gender perspectives into maritime policies and practice.^15^

### Research Gap

Despite these initiatives, women remain significantly underrepresented in the maritime workforce, especially in seagoing roles.^16^ Several factors have been identified, including career barriers, difficult on-board relations, and, most importantly, unsafe or inequitable working conditions.^17^ Ensuring equitable and supportive working conditions is therefore essential for workforce sustainability. In addition, although increasing attention has been paid to seafarers’ working conditions and wellbeing, there is limited research examining how workplace violence and relational dynamics contribute to mental health outcomes in large international crew.

Addressing this knowledge gap is essential for improving psychosocial safety and the long-term sustainability of the maritime industry. Ensuring decent working conditions at sea is critical not only for seafarers’ mental health but also for safe and efficient vessel operations. Therefore, this study investigates the prevalence and occupational determinants of depression, anxiety, and suicidal ideation among seafarers, with a focus on psychosocial working conditions.

## Materials and Methods

### Study Design and Participants

This quantitative cross-sectional survey conducted among seafarers examined the prevalence and risk factors of depression, anxiety, and suicidal ideation across different tanker vessel types. The study was part of the Maritime Culture Lab research initiative, launched in 2024 by Hafnia (a global tanker-vessel shipping company). The reporting of this study follows the Strengthening the Reporting of Observational Studies in Epidemiology (STROBE) guideline (Supplementary file 1).

### Sampling, Recruitment, and Data Collection

As part of this project, an invitation email was distributed to all seafarers employed by Hafnia. The email contained detailed information about the study and a web link directing participants to an online platform where they could provide informed consent and subsequently complete the questionnaire. The online system also ensured participant anonymity. The survey was open for responses from April 4 to June 8, 2025.

The Regional Scientific Ethics Committee for Southern Denmark reviewed and approved the research project. Formal ethics approval was not required, as the survey was fully anonymized, and the data collected were not notifiable under Danish legislation (Committee Act §1; Journal number 20202000-131). The study protocol was approved by the Research & Innovation Office at [blinded for review]. Written informed consent was obtained from all participants prior to participation.

### Study Variables

The outcome variables in this study included anxiety, depression, and suicidal ideation. Anxiety was measured using the Generalized Anxiety Disorder-7 (GAD-7) questionnaire^18^ and depression was measured using the Patient Health Questionnaire-9 (PHQ-9).^19^ Internal consistency was good for both instruments in the present sample, with a Cronbach’s alpha of 0.90 for the GAD-7 and 0.88 for the PHQ-9.

GAD-7 score of 7 or more was defined as indicating elevated anxiety symptoms, based on previous work on Spanish seafarers that showed work-duty difficulties beginning at a score of 7.^20^ PHQ-9 scores of 9 or more were defined as having elevated depression symptoms, which have been previously used to identify depressive symptoms in a Thai seafarer sample.^21^ Response to item 9 of PHQ-9 has been defined as a moderate predictor of subsequent suicide death and therefore used in these analyses.^22^ Suicidal ideation was therefore assessed using item 9 of the PHQ-9, which asks respondents how often they in the last two weeks have experienced thoughts that they would be better off dead or of self-harm. Any endorsement of item 9 of PHQ-9 (i.e., a score of 1 or higher) was classified as indicating suicidal ideation. The PHQ-9 and GAD-7 are validated instruments,^18,19^ and no substantive modifications were made beyond minor language clarification.

The explanatory variables included exposure to workplace violence (bullying, harassment, verbal threat, and physical and sexual assaults) at least once within the latest 12 months and psychosocial working conditions, which were operationalized using the following variables: perceived social support, job satisfaction, coworker relationships, and perceived fairness in workload distribution.

Bullying, harassment, threats of violence, and physical assault were each assessed with a single item asking whether the participant had experienced the respective behavior onboard during the past 12 months (e.g., “Have you been exposed to [bullying/harassment/threats of violence/physical assault] onboard within the last 12 months?”). Sexual assault included unwanted sexual behaviors, assessed through two questions: “Has someone touched you against your will (e.g., groped you and/or held on to you)?” and *“How many times during the last 12 months have you been exposed to the following in connection with your work onboard: someone attempted or actually had oral, anal, or vaginal intercourse with you when you did not want them to?”* Due to low prevalence, these sexual assault items were combined into a single binary variable indicating exposure to any form of sexual assault. Exposure to workplace violence was coded as ‘1’ = No, ‘2’ = Psychological violence only (including exposure to bullying, harassment and verbal threat), and ‘3’ = Psychological, physical and sexual violence.

Perceived social support was measured using the 12-item Multidimensional Scale of Perceived Social Support (MSPSS; score range 12–84) tool, which measures a person’s perception of the level of social support they receive from family and friends.^23^ Mean scores were categorized into ‘Low’ (1.0–2.9), ‘Moderate’ (3.0–5.0), and ‘High’ (5.1–7.0).^23^ Job satisfaction was assessed by asking: *“How satisfied are you with your job as a whole?”* Responses were recorded on a 5-point Likert scale and dichotomized into ‘2’ = Yes (including “very satisfied” and “satisfied”) and ‘1’ = No (including “partly satisfied,” “unsatisfied,” and “very unsatisfied”).

Relationships with coworkers were assessed by asking: *“To what extent would you say there is a good working relationship between you and your fellow crew members?”* Fair distribution of work was measured using the question: “To what extent would you say the workload is distributed fairly among the crew onboard?” Responses of both variables were recorded on a 5-point Likert scale and dichotomized into ‘2’ = Yes (including “to a large extent”, “to a very large extent”, and “somewhat”) and ‘1’ = No (including “not at all,” or “to a small extent”).

The covariates included age, sex (‘man’, ‘woman’, others), years of experience, rank (‘Senior officer’, ‘Junior officer’, ‘Cadet/Trainee/Others’), Work area (‘Bridge’, ‘Engine’, ‘Deck/Galley/Other’), and nature of contract (‘Permanent’, ‘Temporary/Other’). Seven seafarers identified themselves as other genders and due to the very small number, further analysis for this group was not possible.

### Statistical Analysis

The initial analyses involved calculating frequencies, percentages, mean values, and standard deviations to provide an overview of the study population. Logistic regression models were used to examine the independent association between the explanatory variables and anxiety, depression, and suicidal ideation. Odds ratios (OR) with corresponding 95% CI were reported as the measure of association between the explanatory variables and the outcome variables. All statistical analyses were performed using Stata version 18.0 (StataCorp, College Station, TX, USA).

## Results

### Study Participants

A total of 4,871 seafarers received the questionnaire invitation and of these, 1,369 seafarers participated in the survey (response rate = 28%). A total of 323 did not complete the mental health questions, and thus, the final analysis included a sample of 1,046 fully completed questionnaires. The mean age of participants was 36.7 ± 9.3 years, and the average work experience was 13.0 ± 8.9 years. Nearly half of the participants were cadets, trainees, or others (48.3%, n = 505), followed by senior officers (28.0%, n = 293) and junior officers (23.7%, n = 248). In terms of work area, 38.1% (n = 399) worked in the engine department, 36.3% (n = 380) in deck, galley, or other support areas, and 25.5% (n = 267) on the bridge (Table 1).

**Table 1.** General Characteristics of the Study Participants (N=1046)

​	All participants n (%)	Depression n (%)	Anxiety n (%)	Suicidal thoughts n (%)
**Total sample**	1,046	1,046	1,040	1,046
Prevalence (% [95% CI])*	-	7.5 (6.1, 9.3)	9.2 (7.6, 11.2)	4.7 (3.6, 6.1)
**Age (Mean±SD)**	36.7 (±9.3)	32.6 (±7.0)	34.0 (±7.8)	33.3 (±6.7)
**Sex**				
Woman	112 (10.7)	14 (12.5)	13 (11.6)	4 (3.6)
Man	934 (89.3)	65 (7.0)	83 (8.9)	45 (4.8)
**Years of experience (Mean±SD)**	13.0 (±8.9)	9.7 (±7.0)	11.2 (±7.8)	10.3 (±6.8)
**Rank/position**				
Senior Officers	293 (28.0)	15 (5.1)	35 (12.0)	9 (3.1)
Junior Officers	248 (23.7)	27 (10.9)	25 (10.2)	14 (5.6)
Cadet/Trainee/Others	505 (48.3)	37 (7.3)	36 (7.2)	26 (5.1)
**Work area**				
Bridge	267 (25.5)	27 (10.1)	37 (13.9)	12 (4.5)
Engine	399 (38.1)	27 (6.8)	32 (8.1)	22 (5.5)
Deck/Galley/Other	380 (36.3)	25 (6.6)	27 (7.2)	15 (3.9)
**Nature of contract**				
Permanent	380 (36.3)	32 (8.4)	32 (8.4)	21 (5.5)
Temporary/Other	666 (63.7)	47 (7.1)	64 (9.7)	28 (4.2)
**Job satisfaction**				
No	22 (2.1)	5 (22.7)	6 (27.3)	3 (13.6)
Yes	1,024 (97.9)	74 (7.2)	90 (8.8)	46 (4.5)
**Good working relationship with crew members**				
No	32 (3.1)	12 (37.5)	12 (38.7)	8 (25.0)
Yes	1,014 (96.9)	67 (6.6)	84 (8.3)	41 (4.0)
**Fair distribution of workload**				
No	131 (12.5)	27 (20.6)	27 (20.8)	18 (13.7)
Yes	915 (87.5)	52 (5.7)	69 (7.6)	31 (3.4)
**Perceived Social Support**				
Low	51 (4.9)	8 (15.7)	4 (7.8)	4 (7.8)
Medium	219 (20.9)	22 (10.0)	32 (14.7)	16 (7.3)
High	776 (74.2)	49 (6.3)	60 (7.8)	29 (3.7)
**Exposure to violence**				
No	929 (89.6)	52 (5.6)	68 (7.4)	34 (3.7)
Psychological violence only	93 (9.0)	17 (18.3)	17 (18.3)	9 (9.7)
Psychological, physical and sexual violence	15 (1.4)	7 (46.7)	8 (53.3)	4 (26.7)

Data are n (%) for categorical variables and mean ± SD for continuous variables. Deck, galley and others were merged into one category due to smaller number in some cells. Psychological violence includes exposure to bullying, harassment and verbal threat. Seven participants identified as “other” gender (0.7%). Due to the small sample size, they were excluded from regression analyses to avoid unstable estimates.

The majority of participants (63.7%, n = 666) were employed under temporary or other forms of contracts, while 36.3% (n = 380) held permanent positions. Most reported being satisfied with their jobs (97.9%, n = 1,024) and described having good relationships with other crew members (96.9%, n = 1,014). Likewise, 87.5% (n = 915) perceived the distribution of workload as fair, and 74.2% (n = 776) reported high levels of perceived social support.

Exposure to workplace violence was reported by 10.4% (n = 108) of participants, including 9.0% (n = 93) who experienced psychological violence and 1.4% (n = 15) who reported combined psychological and physical or sexual violence. A significant proportion of the participants preferred not to answer the questions about workplace violence, with 2.7% (n=28) for sexual violence, 1.3% (n=14) for physical violence, and 5.5% (n=58) for psychological violence (either bullying or harassment or threats) (Table 2).

**Table 2.** Participants Responses on the Workplace Violence Questions (N=1046)

​	Depression			Anxiety			Suicidal thoughts		
	Total	No	Yes	Total	No	Yes	Total	No	Yes
	N = 1,046	n = 967	n = 79	N = 1,040	n = 944	n = 96	N=1,046	n = 997	n = 49
**Sexual violence**									
No	1,012 (96.7)	940 (92.9)	72 (7.1)	1,006 (96.7)	918 (91.3)	88 (8.7)	1,012 (96.7)	970 (95.8)	42 (4.2)
Yes	6 (0.6)	1 (16.7)	5 (83.3)	6 (0.6)	2 (33.3)	4 (66.7)	6 (0.6)	2 (33.3)	4 (66.7)
Prefer not to answer	28 (2.7)	26 (92.9)	2 (7.1)	28 (2.7)	24 (85.7)	4 (14.3)	28 (2.7)	25 (89.3)	3 (10.7)
**Physical violence**									
No	1,020 (97.5)	950 (93.1)	70 (6.9)	1,014 (97.5)	931 (91.8)	83 (8.2)	1,020 (97.5)	976 (95.7)	44 (4.3)
Yes	12 (1.1)	7 (58.3)	5 (41.7)	12 (1.2)	5 (41.7)	7 (58.3)	12 (1.1)	9 (75.0)	3 (25.0)
Prefer not to answer	14 (1.3)	10 (71.4)	4 (28.6)	14 (1.3)	8 (57.1)	6 (42.9)	14 (1.3)	12 (85.7)	2 (14.3)
**Bullying**									
No	955 (91.3)	898 (94.0)	57 (6.0)	949 (91.2)	877 (92.4)	72 (7.6)	955 (91.3)	915 (95.8)	40 (4.2)
Yes	64 (6.1)	46 (71.9)	18 (28.1)	64 (6.2)	45 (70.3)	19 (29.7)	64 (6.1)	57 (89.1)	7 (10.9)
Prefer not to answer	27 (2.6)	23 (85.2)	4 (14.8)	27 (2.6)	22 (81.5)	5 (18.5)	27 (2.6)	25 (92.6)	2 (7.4)
**Harassment**									
No	961 (91.9)	905 (94.2)	56 (5.8)	955 (91.8)	880 (92.1)	75 (7.9)	961 (91.9)	923 (96.0)	38 (4.0)
Yes	57 (5.4)	39 (68.4)	18 (31.6)	57 (5.5)	43 (75.4)	14 (24.6)	57 (5.4)	49 (86.0)	8 (14.0)
Prefer not to answer	28 (2.7)	23 (82.1)	5 (17.9)	28 (2.7)	21 (75.0)	7 (25.0)	28 (2.7)	25 (89.3)	3 (10.7)
**Threats**									
No	972 (92.9)	907 (93.3)	65 (6.7)	966 (92.9)	889 (92.0)	77 (8.0)	972 (92.9)	932 (95.9)	40 (4.1)
Yes	45 (4.3)	35 (77.8)	10 (22.2)	45 (4.3)	33 (73.3)	12 (26.7)	45 (4.3)	40 (88.9)	5 (11.1)
Prefer not to answer	29 (2.8)	25 (86.2)	4 (13.8)	29 (2.8)	22 (75.9)	7 (24.1)	29 (2.8)	25 (86.2)	4 (13.8)
**Psychological violence (Bullying, Harassment & Threats)**									
No	897 (85.8)	848 (94.5)	49 (5.5)	891 (85.7)	827 (92.8)	64 (7.2)	897 (85.8)	865 (96.4)	32 (3.6)
Yes	91 (8.7)	68 (74.7)	23 (25.3)	91 (8.8)	71 (78.0)	20 (22.0)	91 (8.7)	79 (86.8)	12 (13.2)
Prefer not to answer	58 (5.5)	51 (87.9)	7 (12.1)	58 (5.6)	46 (79.3)	12 (20.7)	58 (5.5)	53 (91.4)	5 (8.6)

### Prevalence of Mental Health Conditions

The prevalence of anxiety, depression, and suicidal ideation among seafarers was 9.2%, 7.5%, and 4.7%, respectively. Women had higher prevalence of both anxiety (11.6% vs. 8.9%) and depression (12.5% vs. 7.0%) compared with men, whereas suicidal ideation was slightly more common among men (4.8%) than women (3.6%). All three mental health conditions were markedly higher among participants exposed to psychological violence only or combined psychological and physical/sexual violence compared with those reporting no violence exposure.

### Factors Associated With Anxiety

Seafarers exposed to psychological violence only (OR = 2.07; 95% CI: 1.08–3.96) or to both psychological and physical/sexual violence (OR = 6.92; 95% CI: 2.11–22.65) were more likely to report anxiety symptoms compared with those not exposed to any violence. In contrast, seafarers reporting good relationships with crew members (OR = 0.29; 95% CI: 0.10–0.78) and high job satisfaction (OR = 0.26; 95% CI: 0.09–0.82) were less likely to report anxiety symptoms compared with those reporting poor crew relationships or job dissatisfaction, respectively. Compared with senior officers, junior officers (OR = 0.26; 95% CI: 0.13–0.54), and cadet/trainee/other positions (OR = 0.19; 95% CI: 0.09–0.43) were less likely to report anxiety symptoms (Table 3).

**Table 3.** Multivariable Logistic Regression Analysis for Factors Associated With Depression, Anxiety and Suicidal Thoughts Among Seafarers (N=1046)

​	Depression	Anxiety	Suicidal thoughts
Characteristics	OR (95% CI)	OR (95% CI)	OR (95% CI)
**Age**	0.96 (0.90, 1.03)	0.97 (0.91, 1.03)	0.96 (0.89, 1.05)
**Sex**			
Woman	*Ref.*	*Ref.*	*Ref.*
Man	0.8 (0.37, 1.72)	1.17 (0.55, 2.50)	2.32 (0.72, 7.51)
**Years of experience**	0.98 (0.90, 1.06)	0.95 (0.89, 1.02)	0.99 (0.90, 1.08)
**Rank/position**			
Senior Officers	*Ref.*	*Ref.*	*Ref.*
Junior Officers	0.96 (0.42, 2.16)	**0.26 (0.13, 0.54)**	0.92 (0.33, 2.53)
Cadet/Trainee/Others	0.64 (0.26, 1.56)	**0.19 (0.09, 0.43)**	1.07 (0.37, 3.05)
**Work area**			
Bridge	*Ref.*	*Ref.*	*Ref.*
Engine	0.79 (0.42, 1.48)	0.62 (0.35, 1.09)	1.23 (0.55, 2.75)
Deck/Galley/Other	0.63 (0.30, 1.35)	0.62 (0.31, 1.22)	0.61 (0.23, 1.65)
**Nature of contract**			
Permanent	*Ref.*	*Ref.*	*Ref.*
Temporary/Other	0.96 (0.56, 1.66)	1.19 (0.71, 1.99)	0.89 (0.46, 1.72)
**Job satisfaction**			
No	*Ref.*	*Ref.*	*Ref.*
Yes	0.40 (0.12, 1.31)	**0.26 (0.09, 0.82)**	0.55 (0.14, 2.19)
**Good working relationship with crew members**			
No	*Ref.*	*Ref.*	*Ref.*
Yes	**0.35 (0.13, 0.95)**	**0.29 (0.10, 0.78)**	0.46 (0.15, 1.40)
**Fair distribution of workload**			
No	*Ref.*	*Ref.*	*Ref.*
Yes	**0.42 (0.23, 0.79)**	0.54 (0.29, 1.01)	**0.39 (0.19, 0.82)**
**Perceived Social Support**			
Low perceived support	*Ref.*	*Ref.*	*Ref.*
Medium perceived support	0.50 (0.18, 1.40)	2.58 (0.72, 9.16)	0.95 (0.26, 3.50)
High perceived support	**0.34 (0.13, 0.85)**	1.26 (0.37, 4.32)	0.56 (0.16, 1.93)
**Exposure to violence**			
No	*Ref.*	*Ref.*	*Ref.*
Psychological violence only	**2.30 (1.18, 4.49)**	**2.07 (1.08, 3.96)**	1.95 (0.84, 4.53)
Psychological, physical and sexual violence	**5.53 (1.64, 18.67)**	**6.92 (2.11, 22.65)**	**4.32 (1.03, 18.17)**

Note. Bold estimates indicate statistically significant associations (p < 0.05). Bold text in variable headings denotes section/variable labels and does not indicate statistical significance.

### Factors Associated With Depression

Seafarers who were exposed to psychological violence only (OR = 2.30; 95% CI: 1.18–4.49), as well as those exposed to both psychological and physical/sexual violence (OR = 5.53; 95% CI: 1.64–18.67), were more likely to report depressive symptoms compared with those reporting no violence. While, seafarers who reported fair workload distribution (OR = 0.42; 95% CI: 0.23–0.79), good relationships with crew members (OR = 0.35; 95% CI: 0.13–0.95), and high perceived social support (OR = 0.34; 95% CI: 0.13–0.85) were less likely to report depressive symptoms compared with those reporting unfair workload, poor crew relationships, or low social support, respectively (Table 3).

### Factors Associated With Suicidal Ideation

Seafarers exposed to both psychological and physical/sexual violence were more likely to report suicidal ideation (OR = 4.32; 95% CI: 1.03–18.17) compared with those who were not exposed to any violence. Seafarers reporting fair workload distribution (OR = 0.39; 95% CI: 0.19–0.82) were less likely to report suicidal ideation compared with those reporting unfair workload distribution (Table 3).

## Discussion

This study revealed a concerning burden of depression, anxiety, and suicidal ideation among seafarers and identified several modifiable occupational factors associated with these outcomes. Seafarers exposed to psychological and physical/sexual violence at their workplace had markedly higher odds of depression, anxiety, and suicidal ideation compared with those unexposed. Fair workload distribution, good relationships with crew members, and strong social support were associated with lower odds of these mental health conditions.

The prevalence of anxiety (9.2%) and depression (7.5%) observed in this study is somewhat lower than that reported in several recent studies of seafarers using validated screening tools, but remains within the range documented in the literature. For example, a multinational study using the PHQ-9 and GAD-7 reported prevalence estimates of 14.1% for depression and 12.4% for anxiety,^24^ while a more recent Canadian study found substantially higher levels (39.4% depression and 31.8% anxiety).^25^ Similarly, recent studies have reported elevated levels of psychological distress among seafarers, including 38.8% for depression and 56.7% for anxiety using standardized instruments such as the Beck Depression Inventory-Second Edition (BDI-II) and GAD-7.^26^

Importantly, our findings regarding the strong association between workplace violence and adverse mental health outcomes are highly consistent with prior seafarer-specific research, which has identified interpersonal stressors, including bullying, social isolation, and hierarchical pressure, as key determinants of depression and anxiety.^27,28^

Research on the prevalence of suicidal ideation is limited, likely due to stigma and shame surrounding the topic. In the present study, suicidal ideation was slightly more common among men (4.8%) than women (3.6%), a pattern consistent with other research findings.^29^ The International Transport Workers’ Federation (ITF) Seafarers’ Trust survey found that 6% to 35% of respondents knew colleagues who had considered suicide, depending on their home country.^2^ The marine insurance company GARD released a study in 2025, reporting that suicides accounted for more deaths than fatal incidents, with officers appearing particularly vulnerable and the risk being higher during the first three months on board than later in the contract.^30^

The results also suggest gender differences in mental health outcomes, with women reporting higher levels of anxiety and depression. This is consistent with evidence from earlier studies showing that women at sea face disproportionate risks linked to isolation, harassment, and lack of peer support, although these studies include crews where females represented a low proportion of the crew.^7,12,13,31^ Compared to men, who were more often employed as senior officers or ratings, women were disproportionately represented on the bridge, with many serving as cadets or trainees. These structural differences in rank and experience may further influence women’s exposure to stress and vulnerability. Crews with higher female representation experience improved work environments and social relationships for both female and male seafarers, indicating that increasing the number of women onboard may have positive effects on crew wellbeing more broadly.^14^

Compared with a previous study (2025),^12^ which reported that 24.4% of participants experienced workplace violence, the prevalence in the present study was substantially lower at 10.4%. However, psychological violence remained the most common form of violence in both studies and exposure to multiple forms of violence was associated with substantially higher mental health risks.

Several studies have demonstrated that competent and supportive officers can significantly improve the work environment by setting standards onboard.^7,10,12,17^ The working environment at sea must be stronger and more supportive than many onshore workplaces to compensate for structural challenges such as prolonged separation from family, confined living spaces, and the inability to detach from work-related stress. Therefore, enhancing leadership quality, ensuring fair workload distribution, strengthening crew relationships, maintaining clear reporting procedures and mental health support channels can all contribute to a healthier and safe work environment for seafarers.

Interventions, such as supervisor training in mental health literacy and self-care, peer mentoring, and structured conflict management, may not only reduce stress and interpersonal tensions but also improve overall morale and retention. The study by Froholdt and Rasmussen (2025) suggests that workplace health interventions onboard merchant vessels may reduce stress by enhancing individual coping resources.^32^ Their findings showed a significant reduction in perceived stress following a multifaceted intervention that combined health promotion, coaching, and workshops.^32^ Evidence from other transport sectors suggests that comparable interventions can reduce psychological distress and enhance team cohesion.^33^ Exploring the adaptation of these approaches to shipboard contexts could support maritime organisations.

Our findings have implications for action across multiple levels of the maritime system. At the international level, the IMO and International Labour Organization (ILO) could strengthen guidance on psychosocial risk management, workplace violence prevention, and mental health monitoring within existing maritime labour and safety framework. Shipping organisations should implement supervisor mental health training, confidential reporting pathways for workplace violence, and structured peer-support programmes, particularly during the first months onboard, when mental health risks may be elevated. Officers and seafarers should promote respectful workplace behaviours, fair workload sharing, and peer support, consistent with the protective role of supportive shipboard environments observed in this study. As psychological safety has become increasingly recognised as a prerequisite for healthy and safe workplaces,^34^ further research is needed to understand how it can be developed and sustained in a maritime context. Such knowledge may better support psychosocial work environment initiatives by strengthening speaking up, learning, collaboration, and ultimately the health, safety, and retention of seafarers.

This study also has limitations. First, distributing the survey through the shipowner may have discouraged participation and honest reporting due to concerns about anonymity: a selection bias affecting who responded and an information bias affecting how they responded. This may also explain the proportion of “prefer not to answer” responses to sensitive questions on workplace violence. Second, the cross-sectional design precludes establishing temporal relationships between covariates and outcomes, and the sequence of exposure, mediator, and outcome cannot be determined. Third, all workplace violence outcomes were assessed using single self-reported items rather than validated multi-item instruments, limiting measurement precision and potentially introducing reporting bias. Fourth, the reliance on self-reported questionnaires may have introduced recall bias. Additionally, no a priori sample size or statistical power calculation was conducted, so the study may have been underpowered to detect some associations. Unmeasured factors, including cultural, physical health, and behavioural characteristics, may also have influenced both exposures and outcomes.^35^ Fifth, attempts to conduct cross-analyses (e.g., stratified or interaction models) to examine differential effects across subgroups were undertaken but could not be reliably completed: several stratified cells contained too few cases, producing unstable estimates and models that did not converge. Finally, the relatively low response rate (28%) may raise concerns about representativeness; however, similar response rates have been reported in comparable maritime studies, supporting the relevance of the findings.^12^

## Conclusions

This study demonstrates that mental health among seafarers is closely tied to the psychosocial quality of the shipboard environment. Anxiety and depression are prevalent, particularly among those exposed to psychological, physical, or sexual violence, while fair workload distribution, strong working relationships, and high perceived social support serve as protective factors. These findings emphasize that addressing preventable stressors and fostering safe, inclusive, and supportive environments, especially within diversity-crewing models, is critical for promoting the wellbeing, resilience, and long-term sustainability of the global seafaring workforce. The results also suggest that enhanced policies and regulations are warranted to reduce the incidence of violence at sea and to promote psychological safety onboard and protect seafarers’ safety and wellbeing.

Future research should establish prospective cohort studies linked to maritime occupational health, safety, and employment records to examine workplace adversities and mental health outcomes over time. These linked registry-based studies would enable robust stratified and interaction analyses across key workforce characteristics, including gender and rank. In addition, intervention studies (pre-post or controlled designs) should evaluate the effectiveness of supervisor mental health training, peer-mentoring programmes, and structured onboard wellbeing interventions, while examining how increased female representation influences organisational culture to inform evidence-based diversity and crewing policies.

## Supplemental Material

Supplemental material - Psychosocial Working Conditions, Workplace Violence, and Mental Health Outcomes Among Seafarers Supplemental material for Psychosocial Working Conditions, Workplace Violence, and Mental Health Outcomes Among Seafarers by Subash Thapa, Lisa Loloma Froholdt, Santosh Giri, and Jesper Bo Nielsen in The Journal of Health Care Organization, Provision, and Financing.

Supplemental material - Psychosocial Working Conditions, Workplace Violence, and Mental Health Outcomes Among Seafarers Supplemental material for Psychosocial Working Conditions, Workplace Violence, and Mental Health Outcomes Among Seafarers by Subash Thapa, Lisa Loloma Froholdt, Santosh Giri, and Jesper Bo Nielsen in The Journal of Health Care Organization, Provision, and Financing.

## Data Availability

The datasets used and/or analysed during the current study available from the corresponding author on reasonable request.*
