# Supplementary material for: Psychosocial Working Conditions, Workplace Violence, and Mental Health Outcomes Among Seafarers
Source: Inquiry. 2026 Sep 7;63:00469580261483999. doi: 10.1177/00469580261483999 (PMC13554583; doi:10.1177/00469580261483999)
Supplement: Supplementary file 2 [file sj-pdf-2-inq-10.1177_00469580261483999.pdf]

Hafnia (Bullying & Harrassment Survey)

Start of Block: Introduction

Q1
MHT Maritime Culture Lab Work Environment and Bullying & Harassment Survey Thank you for taking part in this survey. This questionnaire is about you and the way you see your workplace, and it is part of the Maritime Culture Lab study at Hafnia. There are 7 main sections. Your experiences are important, and we hope you will take time to participate and fill in all the questions. This survey is anonymous, and we follow the strict European GDPR rules on data handling. Thanks again.

End of Block: Introduction

Start of Block: Block 1

Q2 **Section 1: General questions** The first questions are about your employment and your work.

Q3 1A. What part of the company do you work in?

- ○ Bridge (1)
- ○ Deck (2)
- ○ Engine (3)
- ○ Galley (4)
- ○ Other (5)

Q4 1B. Are you?

- ◯ Woman (1)
- ◯ Man (2)
- ◯ Other (3)

Q5 1C. How old are you (years)? *Please enter your age in years.*

▼ 18 (18) ... 99 (99)

Q6 1D. How long have you worked at sea (years)? *Please enter your experience in years below. Enter 0 if less than 1 year.*

▼ 0 (0) ... 99 (99)

Q7 1E. What is your rank/position onboard?

- ◯ Cadet/trainee (1)
- ◯ Rating (2)
- ◯ Junior officer (3)
- ◯ Senior officer (4)
- ◯ Other (5)

Q8 1F. How would you describe your current employment?

◯ I am employed on a permanent contract/shipping company contract (1)

◯ I am employed on a temporary contract/ship contract (2)

◯ I am employed through an agency (3)

◯ I am employed on a voyage contract (4)

◯ Other (5)

Display this question:

If Q8 = 5

Q9 1G. Other (please specify)

____

____

____

____

____

End of Block: Block 1

Start of Block: Block 2

Q10 **Section 2: Work Enviornment**

X→

Q11 2. How satisfied are you with...

	Not at all (0)	To a small extent (1)	Somewhat (2)	To a large extent (3)	To a very large extent (4)
your working conditions? (1)	○	○	○	○	○
your job as a whole? (2)	○	○	○	○	○

End of Block: Block 2

Start of Block: Block 3

Q12 **Section 2: Work Enviornment**

Q13 3. To what extent would you say?

	Not at all (0)	To a small extent (1)	Somewhat (2)	To a large extent (3)	To a very large extent (4)
Your primary senior officer/Team leader is willing to listen to your problems/challenges at work. (1)	○	○	○	○	○
You get help and support from your primary senior officer/team leader. (2)	○	○	○	○	○
The captain makes sure there is a good work environment onboard. (3)	○	○	○	○	○
The workload is distributed fairly among the crew. (4)	○	○	○	○	○
It is ok for crewmembers to ask questions, give an opinion or comment on things onboard. (5)	○	○	○	○	○
Crewmembers share information to each other. (6)	○	○	○	○	○
There is a good work relationship between you and your fellow crewmembers. (7)	○	○	○	○	○

End of Block: Block 3

Start of Block: Block 16

Q14 4. Have you experienced an improvement of the work environment onboard within the last 12 months?

- ◯ Not at all (0)
- ◯ To a small extent (1)
- ◯ Somewhat (2)
- ◯ To a large extent (3)
- ◯ To a very large extent (4)

End of Block: Block 16

Start of Block: Block 4

Q15 **Section 2: Work Enviornment**

Q16 5. Have you sailed on a ship with female seafarers?

- ◯ Yes (1)
- ◯ No (2)

Display this question:

If Q16 = 1

Q17 5A. Have you noticed any changes in the work environment onboard ships with females compared to ships with only males?

- ◯ Yes (1)
- ◯ No (2)

Display this question:

If Q17 = 1

Q18 5A1. If yes, what changes have you noticed?

____

____

____

____

____

Display this question:

If Q16 = 1

Q19 5B. Are there differences in the way the crew communicates?

- ○ Yes (1)
- ○ No (2)

Display this question:

If Q19 = 1

Q68 5B1. If yes, how has the way the crew communicates changed?

____

____

____

____

____

Display this question:

If Q16 = 1

Q20 5C. Are there any changes in lifestyle onboard the ship?

- Yes (1)
- No (2)

Q21 6. If you have anything you would like to add about your work environment onboard, please write it here: *Response should be less than 255 characters including space.*

____

____

____

____

____

End of Block: Block 4

Start of Block: Block 5

Q22 **Section 2: Work Enviornment**

Q23 7. Are there conflicts onboard?

- Not at all (0)
- To a small extent (1)
- Somewhat (2)
- To a large extent (3)
- To a very large extent (4)

Q24 8. Are conflicts handled in a fair way?

- Not at all (0)
- To a small extent (1)
- Somewhat (2)
- To a large extent (3)
- To a very large extent (4)

End of Block: Block 5

Start of Block: Block 6

Q25 **Section 2: Work Enviornment**

Q26 9. Have you been exposed to threats of violence onboard during the last 12 months?

- Yes, daily (1)
- Yes, weekly (2)
- Yes, monthly (3)
- Yes, a few times (4)
- No (5)
- Prefer not to answer (6)

Display this question:

If Q26 = 1

Or Q26 = 2

Or Q26 = 3

Or Q26 = 4

Q27 9A. If yes, by whom? *You can choose more than one.*

- ▢ Colleague (1)
- ▢ Senior officer/Team leader (2)
- ▢ Lower ranking crew (3)
- ▢ Clients/Customers/Passengers (4)
- ▢ Others from the same shipping company (5)

Display this question:

If Q27 = 5

Q28 9B. If others, which category?

____

____

____

____

____

Q29 10. Have you been exposed to physical violence onboard during the last 12 months?

- ○ Yes, daily (1)
- ○ Yes, weekly (2)
- ○ Yes, monthly (3)
- ○ Yes, a few times (4)
- ○ No (5)
- ○ Prefer not to answer (6)

Display this question:

If Q29 = 1

Or Q29 = 2

Or Q29 = 3

Or Q29 = 4

Q30 10A. If yes, by whom? *You can choose more than one.*

- ▢ Colleague (1)
- ▢ Senior officer/Team leader (2)
- ▢ Lower ranking crew (3)
- ▢ Clients/Customers/Passengers (4)
- ▢ Others from the same shipping company (5)

Display this question:

If Q30 = 5

Q31 10B. If others, which category?

____

End of Block: Block 6

Start of Block: Block 7

Q32 **Section 3: Bullying**

Q33 11. Have you been exposed to bullying in your current workplace onboard within the last 12 months?

- ◯ Yes, daily (1)
- ◯ Yes, weekly (2)
- ◯ Yes, monthly (3)
- ◯ Yes, a few times (4)
- ◯ No (5)
- ◯ Prefer not to answer (6)

Display this question:

If Q33 = 1

Or Q33 = 2

Or Q33 = 3

Or Q33 = 4

Q34 12. How long did the bullying go on?

- ◯ Just a few weeks (1)
- ◯ 2 to 3 months (2)
- ◯ 4 to 6 months (3)
- ◯ Between 6 months and 1 year (4)

Display this question:

If Q33 = 1

Or Q33 = 2

Or Q33 = 3

Or Q33 = 4

Q35 13. How many persons bullied you? *If you do not want to answer any of the following, leave as it is.*

- ◯ Number of men (1) ____
- ◯ Number of women (2) ____
- ◯ Number of other persons (3) ____

Display this question:

If Q33 = 1

Or Q33 = 2

Or Q33 = 3

Or Q33 = 4

Q36 14. Who bullied you? *You can choose more than one*

- ☐ Colleague (1)
- ☐ Senior officer/Team leader (2)
- ☐ Lower ranking crew (3)
- ☐ Clients/Customers/Passengers (4)
- ☐ Others from the same shipping company (5)

Display this question:

If Q36 = 5

Q37 14A. Which category?

____

____

____

____

____

Display this question:

If Q33 = 1

Or Q33 = 2

Or Q33 = 3

Or Q33 = 4

Q38 14B. In which way were you bullied most often? *You can choose more than one.*

- ☐ I was hit, kicked, pushed, shoved around, or locked indoors (1)
- ☐ I was made fun of because of my race, or colour (2)
- ☐ I was made fun of because of my religion (3)
- ☐ I was made fun of with sexual jokes, comments, or gestures (4)
- ☐ I was left out of activities on purpose or completely ignored (5)
- ☐ I was made fun of because of how my body or face looked (6)
- ☐ I was bullied in some other way (Specify) (7)

Display this question:

If Q38 = 7

Q39 14C. Other way (Specify)

____

____

____

____

____

Q40 15. Have you been bullied in the previous workplace?

- Yes, daily (1)
- Yes, weekly (2)
- Yes, monthly (3)
- Yes, a few times (4)
- No (5)
- Prefer not to answer (6)

End of Block: Block 7

Start of Block: Block 8

Q41 **Section 3: Bullying**

Q42 16. Which of the following unwanted actions or negative situations have you experienced at your workplace onboard within the last 12 months?

	Daily (1)	Weekly (2)	Monthly (3)	Now and then (4)	Never (5)
16A. Necessary information was withheld from you, making your work difficult (3)	○	○	○	○	○
16B. Gossip and rumours were spread about you (1)	○	○	○	○	○
16C. You were ignored or excluded from the social side of the team (2)	○	○	○	○	○
16D. You were criticised as a person, e.g., due to your habits or background, your opinions (4)	○	○	○	○	○
16E. You were shouted at or made the target of spontaneous anger (5)	○	○	○	○	○
16F. You were repeatedly reminded of your errors or mistakes (6)	○	○	○	○	○
16G. You were met with	○	○	○	○	○

hostility or silence as a response to a question or an attempt to (7)					
16H. Your work and effort were persistently criticised (8)	○	○	○	○	○
16I. Inappropriate jokes were made at your expense by people you don't get along with (9)	○	○	○	○	○
16J. You were subjected to excessive teasing and joking (10)	○	○	○	○	○

End of Block: Block 8

Start of Block: Block 9

Q43 **Section 4: Harassment** The next questions are about harassment.

X→

Q44 17. Have you been exposed to harassment at your current workplace onboard during the last 12 months?

- ○ Yes, daily (1)
- ○ Yes, weekly (2)
- ○ Yes, monthly (3)
- ○ Yes, a few times (4)
- ○ No (5)
- ○ Prefer not to answer (6)

Display this question:

If Q44 = 1

Or Q44 = 2

Or Q44 = 3

Or Q44 = 4

Q45 18. If yes, by whom? *You can choose more than one*

- ☐ Colleague (1)
- ☐ Senior officer/team leader (2)
- ☐ Lower ranking crew (3)
- ☐ Clients/customers/passengers (4)
- ☐ Others from the same shipping company (5)
- ☐ Prefer not to answer (6)

Display this question:

If Q45 = 5

Q46 18A. Which category?

____

____

____

____

____

Display this question:

If Q44 = 1

Or Q44 = 2

Or Q44 = 3

Or Q44 = 4

Q47 19. If yes, which of the following was the reason for the harassment? You can choose more than one

- ▢ Race (1)
- ▢ Religion or belief (2)
- ▢ Cultural background (3)
- ▢ Sex/Gender (4)
- ▢ Sexual orientation (5)
- ▢ Other (which reason) (6)

Display this question:

If Q47 = 6

Q48 19A. Which reasons (specify)?

____

____

____

____

____

End of Block: Block 9

Start of Block: Block 15

Q49 20. How many times during the last 12 months have you been exposed to the following in connection with your work onboard?

	Once (1)	2 to 5 times (2)	6 to 9 times (3)	10 times or more (4)	Never (5)	Prefer not to say (6)
20A. Someone making unwanted sexual comments about your body, clothes or lifestyle (1)	○	○	○	○	○	○
20B. Someone making unwanted sexual comments in a larger group/gathering (4)	○	○	○	○	○	○
20C. Someone sending you unwanted messages with sexual content (e.g., letters, text messages, e-mails or messages on social media (2)	○	○	○	○	○	○
20D. Someone staring or flirtatious glances with sexual undertones that were unwanted or unpleasant to you (3)	○	○	○	○	○	○
20E. Someone touching or fondling you in a sexual way when you did not want them	○	○	○	○	○	○

to (5)						
20F. Someone forcing you to touch their body in a sexual way when you did not want them to (6)	○	○	○	○	○	○
20G. Someone attempting oral, anal, or vaginal intercourse with you when you did not want them to (7)	○	○	○	○	○	○
20H. Someone actually had oral, anal, or vaginal intercourse with you when you did not want them to (8)	○	○	○	○	○	○

End of Block: Block 15

Start of Block: Block 10

Q50 **Section 5: Witnessing Bullying and Harassment Onboard and Support** The next questions are about whether you have witnessed bullying and harassment onboard and support.

Q51 21. Within the past 12 months, have you seen a colleague being bullied or harassed in your current workplace onboard?

- Yes (1)
- No (2)
- Don’t know (3)
- Prefer not to answer (4)

Display this question:

If Q51 = 1

Q52 21A. Which position did the colleague, and the offender have?

- The offender had a higher rank than the colleague (1)
- The offender and the colleague had the same rank (2)
- The offender had a lower rank than the colleague (3)
- Don’t know (4)
- Prefer not to answer (5)

Q53 22. Within the past 12 months, have you seen a colleague being subjected to unwanted sexual attention or sexual harassment at your current workplace onboard?

- Yes (1)
- No (2)
- Don’t know (3)
- Prefer not to answer (4)

Display this question:

If Q53 = 1

Q54 22A. If you said yes to questions 21 and 22, which position did the colleague, and the offender have?

- ◯ The offender had a higher rank than the colleague (1)
- ◯ The offender and the colleague had the same rank (2)
- ◯ The offender had a lower rank than the colleague (3)
- ◯ Don't know (4)
- ◯ Prefer not to answer (5)

Display this question:

If Q51 = 1

Or Q53 = 1

Q55 23. If you said yes to question 21 or 22, how often have you seen it within the last 12 months?

- ◯ Once (1)
- ◯ Few times (2)
- ◯ Monthly (3)
- ◯ Weekly (4)
- ◯ Daily (5)
- ◯ Don't know (6)
- ◯ Prefer not to answer (7)

End of Block: Block 10

Start of Block: Block 11

Q56 **Section 5: Witnessing Bullying and Harassment Onboard and Support** The next questions are about efforts made to address bullying and harassment.

Q57 24. Are you aware of the existing support measures?

	To a large extent (1)	Somewhat (2)	To a small extent (3)	Not at all (4)	Don't know (5)
24A. Have you received information from your company on how to report if you have or a colleague has experienced bullying or harassment? (1)	○	○	○	○	○
24B. Have you taken part in training on bullying and harassment as a part of a training program, e.g., for safety organisation members onboard the ship? (2)	○	○	○	○	○
24C. Are you aware of the hotline that is available to you if you have experienced or witnessed bullying or harassment? (3)	○	○	○	○	○

Page Break

Q58 25. Have you?

	To a large extent (1)	Somewhat (2)	To a small extent (3)	Not at all (4)	Don't know (5)
25A. Noticed a change in relation to bullying and harassment in the Hafnia Fleet over the last 5 years? (1)	○	○	○	○	○
25B. Noticed that this change has improved the work environment in the Hafnia Fleet? (2)	○	○	○	○	○

Display this question:

If Q58 [1] (Count) > 0

Or Q58 [2] (Count) > 0

Or Q58 [3] (Count) > 0

Q59 25C. If you answered yes to any of the above questions, which changes have been most successful in your opinion?

____

____

____

____

____

Q60 26. Do you need any more information on bullying and harassment?

- ○ Yes (1)
- ○ No (2)
- ○ Don’t know (3)

End of Block: Block 11

Start of Block: Block 12

Q61 **Section 6: Social Support**

Q62 28. We are interested in how you feel about the following statements. Read each statement carefully. Indicate how you feel about each statement.

	Very Strongly Disagree (1)	Strongly Disagree (2)	Mildly Disagree (3)	Neutral (4)	Mildly Agree (5)	Strongly Agree (6)	Very Strongly Agree (7)
28A. My family really tries to help me. (1)	○	○	○	○	○	○	○
28B. I can talk about my problems with my family. (2)	○	○	○	○	○	○	○
28C. My family is willing to help me make decisions. (3)	○	○	○	○	○	○	○
28D. I get the emotional help and support I need from my family. (4)	○	○	○	○	○	○	○
28E. My friends really try to help me. (5)	○	○	○	○	○	○	○
28F. I can count on my friends when things go wrong. (6)	○	○	○	○	○	○	○

28G. I have friends with whom I can share my joys and sorrows. (7)	○	○	○	○	○	○	○
28H. I can talk about my problems with my friends. (8)	○	○	○	○	○	○	○
28I. There is a special person in my life who cares about my feelings. (9)	○	○	○	○	○	○	○
28J. There is a special person who is around when I am in need. (10)	○	○	○	○	○	○	○
28K. There is a special person with whom I can share my joys and sorrows. (11)	○	○	○	○	○	○	○

28L. I have a special person who is a real source of comfort to me. (12)	○	○	○	○	○	○	○

End of Block: Block 12

Start of Block: Block13

Q63 **Section 7: Mental Health**

X→

Q64 29. Over the last two weeks, how often have you been feeling the following:

	None (0)	A little bit (1)	Most of the time (2)	All the time (3)
29A. Feeling slack, not wanted to do anything? (1)	○	○	○	○
29B. Feeling unhappy, depressed, really no good, that your spirit was sad? (2)	○	○	○	○
29C. Found it hard to sleep at night, or had other problems with sleeping? (3)	○	○	○	○
29D. Feeling tired or weak, that you have no energy? (4)	○	○	○	○
29E. Poor appetite or overeating (5)	○	○	○	○
29F. Feeling bad about yourself, that you are useless, no good, that you have let your family down? (6)	○	○	○	○
29G. Feeling like you can't think straight or clearly, it's hard to learn new things or concentrate? (7)	○	○	○	○
29H. Been talking slowly or moving around slow? Or the opposite, feeling	○	○	○	○

that you can't sit still; you keep moving around too much? (8)				
29I. Been thinking about hurting yourself or killing yourself? (9)	○	○	○	○

End of Block: Block13

Start of Block: Block 14

Q65 **Section 7: Mental Health**

Q66 30. In the last two weeks, how often you have been bothered by the following problems:

	None (1)	A little bit (8)	Most of the time (9)	All the time (10)
30A. Feeling nervous, anxious, or on edge (1)	○	○	○	○
30B. Being unable to control excessive worrying or maladaptive thoughts (2)	○	○	○	○
30C. Worrying too much about different things (3)	○	○	○	○
30D. Having trouble relaxing (4)	○	○	○	○
30E. Feeling restless (5)	○	○	○	○
30F. Becoming annoyed or irritable (6)	○	○	○	○
30G. Feeling as though something bad might happen (7)	○	○	○	○

Page Break

Q67 31. In general, how would you rate the status of your health? *Rating: 0 to 10*

- 0 (0)
- 1 (1)
- 2 (2)
- 3 (3)
- 4 (4)
- 5 (5)
- 6 (6)
- 7 (7)
- 8 (8)
- 9 (9)
- 10 (10)

End of Block: Block 14
